# 3,8-Dimethyl­acenaphthyl­ene-1,2-dione

**DOI:** 10.1107/S1600536811028479

**Published:** 2011-07-23

**Authors:** Shu-hua He

**Affiliations:** a School of Chemistry and Chemical Engineering, Yangtze Normal University, Fuling 408100, Chongqing, People’s Republic of China

## Abstract

In the title compound, C_14_H_10_O_2_, the acenaphthene­quinone core is essentially planar, with an r.m.s. deviation of 0.0140 Å. In the crystal, mol­ecules are connected by π–π stacking inter­actions [centroid–centroid distances = 3.766 (3), 3.839 (3) and 3.857 (3) Å], forming columns parallel to the *a* axis.

## Related literature

For the synthesis and applications of corannulene (systematic name: dibenzo[*ghi*,*mno*]fluoranthene) and its derivatives, see: Wu & Siegel (2006 ▶); Tsefrikas & Scott (2006 ▶); Sygula (2011 ▶); Zabula *et al.* (2011 ▶); Barth & Lawton (1966 ▶); Scott *et al.* (1997 ▶). For the synthesis of the title compound, see: Guillermet *et al.* (2009 ▶); Seiders *et al.* (1999 ▶); Mori *et al.* (2007 ▶). For the structure of related compounds, see: Abdourazak *et al.* (1994 ▶); Mochida & Yoza (2010 ▶).
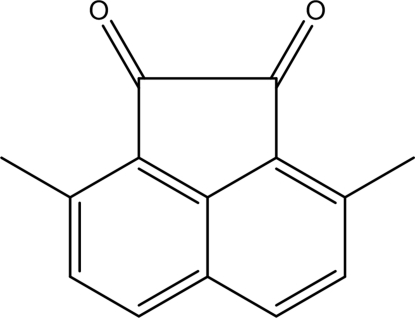


## Experimental

### 

#### Crystal data

C_14_H_10_O_2_
*M* *_r_* = 210.22
Triclinic, 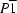

*a* = 7.5415 (8) Å
*b* = 8.5562 (11) Å
*c* = 9.8925 (12) Å
α = 67.891 (11)°
β = 88.310 (9)°
γ = 63.998 (11)°
*V* = 524.45 (13) Å^3^
*Z* = 2
Mo *K*α radiation
μ = 0.09 mm^−1^
*T* = 293 K
0.42 × 0.35 × 0.35 mm

#### Data collection

Agilent Xcalibur Eos diffractometer
Absorption correction: multi-scan (*CrysAlis PRO*; Agilent, 2010 ▶) *T*_min_ = 0.964, *T*_max_ = 1.000
3865 measured reflections
1844 independent reflections
1247 reflections with *I* > 2σ(*I*)
*R*_int_ = 0.021

#### Refinement

*R*[*F* ^2^ > 2σ(*F* ^2^)] = 0.052
*wR*(*F* ^2^) = 0.148
*S* = 1.09
1844 reflections
147 parameters
H-atom parameters constrained
Δρ_max_ = 0.17 e Å^−3^
Δρ_min_ = −0.17 e Å^−3^

### 

Data collection: *CrysAlis PRO* (Agilent, 2010 ▶); cell refinement: *CrysAlis PRO*; data reduction: *CrysAlis PRO*; program(s) used to solve structure: *SHELXS97* (Sheldrick, 2008 ▶); program(s) used to refine structure: *SHELXL97* (Sheldrick, 2008 ▶); molecular graphics: *OLEX2* (Dolomanov *et al.*, 2009 ▶); software used to prepare material for publication: *OLEX2*.

## Supplementary Material

Crystal structure: contains datablock(s) global, I. DOI: 10.1107/S1600536811028479/rz2626sup1.cif

Structure factors: contains datablock(s) I. DOI: 10.1107/S1600536811028479/rz2626Isup2.hkl

Supplementary material file. DOI: 10.1107/S1600536811028479/rz2626Isup3.cml

Additional supplementary materials:  crystallographic information; 3D view; checkCIF report

## Figures and Tables

**Fig. 1.**
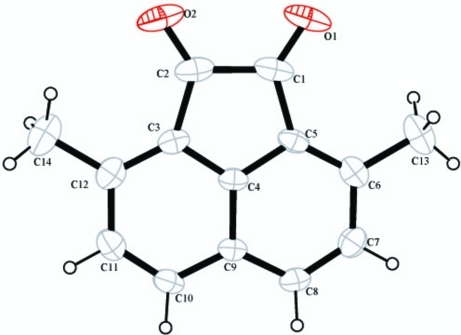
The molecular structure of the title compound, with displacement ellipsoids drawn at the 30% probability level.

**Fig. 2.**
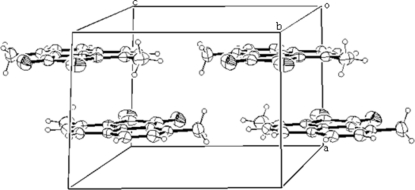
Packing diagram of the title compound. Displacement ellipsoids are drawn at the 30% probability level.

## supplementary crystallographic information

### Comment

Corannulene (IUPAC name: dibenzo[*ghi*,*mno*]fluoranthene) and its derivatives have triggered intense interest due to their unusual bowl-formed structure (Wu & Siegel, 2006; Tsefrikas & Scott, 2006). Recently, a number of novel corannulene derivatives with excellent optical properties have been reported (Sygula, 2011; Zabula *et al.*, 2011). Up to now, different synthetic methods have been reported (Barth & Lawton, 1966; Scott *et al.*, 1997). The title compound, the synthesis of which has been reported in literatures (Guillermet *et al.*, 2009; Seiders *et al.*, 1999; Mori *et al.*, 2007), is an important intermediate in synthesizing corannulene and its derivatives. Based on the importance of the relationship between structures and properties, the crystal structure of the title compound is reported herein.

The title molecule (Fig. 1) possesses a *pseudo*-mirror plane and is substantially planar, with a r.m.s. deviation of 0.0140 Å and maximum displacement of 0.026 (3) Å for atom C8. The dihedral angles between the C1–C5 five membered ring and the C3–C4/C9–C12 and C4–C9 benzene rings are 1.42 (9) and 0.81 (9)°, respectively. The C13–C6–C5–C1, C13–C6–C7–C8, C14–C12–C3–C2 and C14–C12–C11-C10 torsion angles are 3.6 (5), 178.4 (3), 0.4 (5) and 178.5 (4)°, respectively. As already observed in related compounds (Abdourazak *et al.*, 1994; Mochida & Yoza, 2010), the C1–C2 bond length (1.569 (4) Å) is remarkably longer than expected for a C(*sp*^2)– C(^*^sp^*2) bond, and the bond angles involving the carbonyl groups ( O1—C1—C5 = 130.6 (3)°, O1—C1—C2 = 123.5 (2)°, C6—C5—C1 = 133.3 (2)°) are larger than the ideal value of 120°. In the crystal (Fig. 2), molecules are linked into columns parallel to the *a* axis by π–π stacking interactions [Cg1···Cg2^i^ = 3.766 (3) Å; Cg2···Cg2^i^ = 3.839 (3) Å; Cg2···Cg2^ii^ = 3.857 (3) Å; Cg1 and Cg2 are the centroids of the C3–C4/C9–C12 and C4–C9 rings, respectively. Symmetry codes: (i) 2-x, 1-y, 2-z; (ii) 1-x, 1-y, 2-z].

### Experimental

To a mixture of aluminium tribromide (10 g, 37.5 mmol) in CH_2_Cl_2_ (200 ml) was added dropwise a solution of 2,7-dimethylnaphthalene (2.6 g, 16.6 mmol) and oxalylchloride (2.2 ml, 26.0 mmol) in CH_2_Cl_2_ (150 ml) at about 253 K over 15 min. The mixture was stirred vigously at 253 K for additional 6 h and then quenched carefully by pouring into 500 ml ice water. After 30 min, the organic layer was washed with water, dried over MgSO_4_, filtered and evaporated. The residue was dissolved in a minimal amount of CH_2_Cl_2_ and eluted with CH_2_Cl_2_/hexane (1:1 *v*/*v*) though an alumina column to give the title compound as a bright yellow solid (1.26 g, 37% yield, m.p. 478–480 K). Bright yellow single crystals suitable for X-ray diffraction were obtained at room temperature by slow evaporation of a CH_2_Cl_2_ solution over a period of several days.

### Refinement

All H atoms were positioned geometrically (C—H = 0.93–0.96 Å) and refined using a riding model, with *U*_iso_(H) = 1.2*U*_eq_(C) or *U*_iso_(H) = 1.5*U*_eq_(C) for methyl H atoms.

### Crystal data

**Table:**

C_14_H_10_O_2_	*Z* = 2
*M**_r_* = 210.22	*F*(000) = 220
Triclinic, *P*1	*D*_x_ = 1.331 Mg m^−^^3^
Hall symbol: -P 1	Mo *K*α radiation, λ = 0.7107 Å
*a* = 7.5415 (8) Å	Cell parameters from 1026 reflections
*b* = 8.5562 (11) Å	θ = 2.9–29.4°
*c* = 9.8925 (12) Å	µ = 0.09 mm^−^^1^
α = 67.891 (11)°	*T* = 293 K
β = 88.310 (9)°	Block, yellow
γ = 63.998 (11)°	0.42 × 0.35 × 0.35 mm
*V* = 524.45 (13) Å^3^	

### Data collection

**Table:**

Agilent Xcalibur Eos diffractometer	1844 independent reflections
Radiation source: Enhance (Mo) X-ray Source	1247 reflections with *I* > 2σ(*I*)
graphite	*R*_int_ = 0.021
ω scans	θ_max_ = 25.0°, θ_min_ = 2.9°
Absorption correction: multi-scan (*CrysAlis PRO*; Agilent, 2010)	*h* = −8→8
*T*_min_ = 0.964, *T*_max_ = 1.000	*k* = −9→10
3865 measured reflections	*l* = −11→11

### Refinement

**Table:**

Refinement on *F*^2^	Primary atom site location: structure-invariant direct methods
Least-squares matrix: full	Secondary atom site location: difference Fourier map
*R*[*F*^2^ > 2σ(*F*^2^)] = 0.052	Hydrogen site location: inferred from neighbouring sites
*wR*(*F*^2^) = 0.148	H-atom parameters constrained
*S* = 1.09	*w* = 1/[σ^2^(*F*_o_^2^) + (0.0592*P*)^2^ + 0.102*P*] where *P* = (*F*_o_^2^ + 2*F*_c_^2^)/3
1844 reflections	(Δ/σ)_max_ < 0.001
147 parameters	Δρ_max_ = 0.17 e Å^−^^3^
0 restraints	Δρ_min_ = −0.17 e Å^−^^3^

### Special details

**Table:**

Geometry. All e.s.d.'s (except the e.s.d. in the dihedral angle between two l.s. planes) are estimated using the full covariance matrix. The cell e.s.d.'s are taken into account individually in the estimation of e.s.d.'s in distances, angles and torsion angles; correlations between e.s.d.'s in cell parameters are only used when they are defined by crystal symmetry. An approximate (isotropic) treatment of cell e.s.d.'s is used for estimating e.s.d.'s involving l.s. planes.
Refinement. Refinement of *F*^2^ against ALL reflections. The weighted *R*-factor *wR* and goodness of fit *S* are based on *F*^2^, conventional *R*-factors *R* are based on *F*, with *F* set to zero for negative *F*^2^. The threshold expression of *F*^2^ > σ(*F*^2^) is used only for calculating *R*-factors(gt) *etc*. and is not relevant to the choice of reflections for refinement. *R*-factors based on *F*^2^ are statistically about twice as large as those based on *F*, and *R*- factors based on ALL data will be even larger.

### Fractional atomic coordinates and isotropic or equivalent isotropic displacement parameters (Å^2^)

**Table:**

	*x*	*y*	*z*	*U*_iso_*/*U*_eq_	
O1	0.7750 (3)	0.0525 (3)	1.0053 (3)	0.0920 (7)	
O2	0.7510 (3)	0.2516 (3)	0.6896 (3)	0.0968 (8)	
C1	0.7662 (3)	0.2086 (3)	0.9497 (3)	0.0640 (8)	
C2	0.7529 (3)	0.3165 (4)	0.7793 (3)	0.0652 (7)	
C3	0.7426 (3)	0.4999 (3)	0.7582 (3)	0.0507 (6)	
C4	0.7488 (3)	0.5024 (3)	0.8994 (2)	0.0425 (5)	
C5	0.7647 (3)	0.3356 (3)	1.0170 (3)	0.0480 (6)	
C6	0.7721 (3)	0.3212 (3)	1.1597 (3)	0.0545 (7)	
C7	0.7594 (3)	0.4792 (4)	1.1814 (3)	0.0584 (7)	
H7	0.7629	0.4727	1.2774	0.070*	
C8	0.7422 (3)	0.6419 (3)	1.0686 (3)	0.0531 (6)	
H8	0.7337	0.7421	1.0895	0.064*	
C9	0.7374 (3)	0.6586 (3)	0.9216 (3)	0.0446 (6)	
C10	0.7241 (3)	0.8142 (3)	0.7938 (3)	0.0547 (6)	
H10	0.7177	0.9209	0.8026	0.066*	
C11	0.7205 (4)	0.8089 (4)	0.6583 (3)	0.0616 (7)	
H11	0.7125	0.9134	0.5769	0.074*	
C12	0.7283 (3)	0.6538 (4)	0.6344 (3)	0.0592 (7)	
C13	0.7965 (4)	0.1452 (4)	1.2891 (3)	0.0820 (9)	
H13A	0.7773	0.1705	1.3765	0.123*	
H13B	0.9284	0.0439	1.3032	0.123*	
H13C	0.6992	0.1088	1.2703	0.123*	
C14	0.7185 (5)	0.6599 (5)	0.4806 (3)	0.0916 (10)	
H14A	0.7763	0.7373	0.4208	0.137*	
H14B	0.5815	0.7129	0.4380	0.137*	
H14C	0.7915	0.5333	0.4849	0.137*	

### Atomic displacement parameters (Å^2^)

**Table:**

	*U*^11^	*U*^22^	*U*^33^	*U*^12^	*U*^13^	*U*^23^
O1	0.0767 (13)	0.0459 (11)	0.160 (2)	−0.0320 (10)	0.0113 (13)	−0.0430 (12)
O2	0.0918 (15)	0.0941 (15)	0.1333 (19)	−0.0328 (12)	0.0040 (13)	−0.0859 (16)
C1	0.0407 (13)	0.0435 (14)	0.112 (2)	−0.0185 (11)	0.0053 (14)	−0.0368 (15)
C2	0.0447 (14)	0.0609 (16)	0.102 (2)	−0.0178 (12)	0.0048 (13)	−0.0525 (17)
C3	0.0407 (12)	0.0524 (14)	0.0655 (16)	−0.0182 (11)	0.0054 (11)	−0.0340 (13)
C4	0.0320 (11)	0.0403 (12)	0.0601 (15)	−0.0167 (9)	0.0068 (10)	−0.0251 (11)
C5	0.0362 (12)	0.0374 (12)	0.0707 (17)	−0.0182 (10)	0.0073 (11)	−0.0204 (12)
C6	0.0374 (13)	0.0507 (14)	0.0635 (17)	−0.0185 (11)	0.0077 (11)	−0.0134 (12)
C7	0.0515 (14)	0.0681 (17)	0.0553 (16)	−0.0239 (13)	0.0090 (12)	−0.0292 (14)
C8	0.0517 (14)	0.0504 (14)	0.0665 (17)	−0.0219 (12)	0.0078 (12)	−0.0345 (13)
C9	0.0379 (11)	0.0395 (12)	0.0596 (15)	−0.0178 (9)	0.0055 (10)	−0.0233 (11)
C10	0.0543 (14)	0.0414 (13)	0.0690 (18)	−0.0252 (11)	0.0020 (12)	−0.0186 (12)
C11	0.0575 (15)	0.0549 (15)	0.0617 (18)	−0.0273 (13)	0.0039 (12)	−0.0108 (13)
C12	0.0447 (14)	0.0706 (17)	0.0581 (16)	−0.0205 (12)	0.0043 (11)	−0.0289 (14)
C13	0.0662 (18)	0.0680 (18)	0.080 (2)	−0.0287 (15)	0.0118 (15)	−0.0004 (16)
C14	0.087 (2)	0.114 (3)	0.064 (2)	−0.031 (2)	0.0055 (16)	−0.0442 (19)

### Geometric parameters (Å, °)

**Table:**

O1—C1	1.209 (3)	C8—H8	0.9300
O2—C2	1.214 (3)	C8—C9	1.407 (3)
C1—C2	1.569 (4)	C9—C10	1.416 (3)
C1—C5	1.468 (3)	C10—H10	0.9300
C2—C3	1.470 (3)	C10—C11	1.360 (3)
C3—C4	1.407 (3)	C11—H11	0.9300
C3—C12	1.384 (3)	C11—C12	1.410 (3)
C4—C5	1.419 (3)	C12—C14	1.506 (4)
C4—C9	1.401 (3)	C13—H13A	0.9600
C5—C6	1.370 (3)	C13—H13B	0.9600
C6—C7	1.410 (3)	C13—H13C	0.9600
C6—C13	1.504 (3)	C14—H14A	0.9600
C7—H7	0.9300	C14—H14B	0.9600
C7—C8	1.372 (3)	C14—H14C	0.9600
			
O1—C1—C2	123.5 (2)	C4—C9—C8	116.2 (2)
O1—C1—C5	130.6 (3)	C4—C9—C10	116.3 (2)
C5—C1—C2	105.9 (2)	C8—C9—C10	127.5 (2)
O2—C2—C1	123.5 (2)	C9—C10—H10	119.7
O2—C2—C3	130.3 (3)	C11—C10—C9	120.5 (2)
C3—C2—C1	106.2 (2)	C11—C10—H10	119.7
C4—C3—C2	106.6 (2)	C10—C11—H11	118.2
C12—C3—C2	133.0 (2)	C10—C11—C12	123.6 (2)
C12—C3—C4	120.4 (2)	C12—C11—H11	118.2
C3—C4—C5	114.79 (19)	C3—C12—C11	116.6 (2)
C9—C4—C3	122.5 (2)	C3—C12—C14	122.7 (2)
C9—C4—C5	122.7 (2)	C11—C12—C14	120.7 (3)
C4—C5—C1	106.5 (2)	C6—C13—H13A	109.5
C6—C5—C1	133.3 (2)	C6—C13—H13B	109.5
C6—C5—C4	120.2 (2)	C6—C13—H13C	109.5
C5—C6—C7	116.9 (2)	H13A—C13—H13B	109.5
C5—C6—C13	122.5 (2)	H13A—C13—H13C	109.5
C7—C6—C13	120.6 (2)	H13B—C13—H13C	109.5
C6—C7—H7	118.3	C12—C14—H14A	109.5
C8—C7—C6	123.5 (2)	C12—C14—H14B	109.5
C8—C7—H7	118.3	C12—C14—H14C	109.5
C7—C8—H8	119.7	H14A—C14—H14B	109.5
C7—C8—C9	120.5 (2)	H14A—C14—H14C	109.5
C9—C8—H8	119.7	H14B—C14—H14C	109.5
			
O1—C1—C2—O2	−0.2 (4)	C4—C3—C12—C14	−179.2 (2)
O1—C1—C2—C3	179.3 (2)	C4—C5—C6—C7	1.2 (3)
O1—C1—C5—C4	−179.0 (2)	C4—C5—C6—C13	−177.7 (2)
O1—C1—C5—C6	−0.1 (4)	C4—C9—C10—C11	0.7 (3)
O2—C2—C3—C4	179.5 (3)	C5—C1—C2—O2	179.9 (2)
O2—C2—C3—C12	−0.2 (4)	C5—C1—C2—C3	−0.5 (2)
C1—C2—C3—C4	0.0 (2)	C5—C4—C9—C8	−0.1 (3)
C1—C2—C3—C12	−179.7 (2)	C5—C4—C9—C10	179.39 (18)
C1—C5—C6—C7	−177.6 (2)	C5—C6—C7—C8	−0.6 (3)
C1—C5—C6—C13	3.5 (4)	C6—C7—C8—C9	−0.4 (3)
C2—C1—C5—C4	0.8 (2)	C7—C8—C9—C4	0.7 (3)
C2—C1—C5—C6	179.7 (2)	C7—C8—C9—C10	−178.7 (2)
C2—C3—C4—C5	0.6 (2)	C8—C9—C10—C11	−179.8 (2)
C2—C3—C4—C9	−178.55 (18)	C9—C4—C5—C1	178.21 (18)
C2—C3—C12—C11	179.6 (2)	C9—C4—C5—C6	−0.9 (3)
C2—C3—C12—C14	0.4 (4)	C9—C10—C11—C12	0.4 (4)
C3—C4—C5—C1	−1.0 (2)	C10—C11—C12—C3	−0.7 (4)
C3—C4—C5—C6	179.97 (18)	C10—C11—C12—C14	178.5 (2)
C3—C4—C9—C8	178.99 (19)	C12—C3—C4—C5	−179.64 (19)
C3—C4—C9—C10	−1.5 (3)	C12—C3—C4—C9	1.2 (3)
C4—C3—C12—C11	0.0 (3)	C13—C6—C7—C8	178.3 (2)

## References

[bb1] Abdourazak, A. H., Marcinow, Z., Folsom, H. E., Fronczek, F. R., Sygula, R., Sygula, A. & Rabideau, P. W. (1994). *Tetrahedron Lett.* **35**, 3857–3860.

[bb2] Agilent (2010). *CrysAlis PRO* Agilent Technologies, Yarnton, England.

[bb3] Barth, W. E. & Lawton, R. G. (1966). *J. Am. Chem. Soc.* **88**, 380–381.

[bb4] Dolomanov, O. V., Bourhis, L. J., Gildea, R. J., Howard, J. A. K. & Puschmann, H. (2009). *J. Appl. Cryst.* **42**, 339–341.

[bb5] Guillermet, O., Niemi, E., Nagarajan, S., Bouju, X., Martrou, D., Gourdon, A. & Gauthier, S. (2009). *Angew. Chem. Int. Ed.* **48**, 1970–1973. 10.1002/anie.200805689 19191274

[bb6] Mochida, T. & Yoza, K. (2010). *J. Organomet. Chem.* **695**, 1749–1752.

[bb7] Mori, T., Grimme, S. & Inoue, Y. (2007). *J. Org. Chem.* **72**, 6998–7010. 10.1021/jo071216n 17665959

[bb8] Scott, L. T., Cheng, P. C., Hashemi, M. M., Bratcher, M. S., Meyer, D. T. & Warren, H. B. (1997). *J. Am. Chem. Soc.* **119**, 10963–10968.

[bb9] Seiders, T. J., Elliott, E. L., Grube, G. H. & Siegel, J. S. (1999). *J. Am. Chem. Soc.* **121**, 7804–7813.

[bb10] Sheldrick, G. M. (2008). *Acta Cryst.* A**64**, 112–122. 10.1107/S0108767307043930 18156677

[bb11] Sygula, A. (2011). *Eur. J. Org. Chem.* **22**, 1611–1625.

[bb12] Tsefrikas, V. M. & Scott, L. T. (2006). *Chem. Rev.* **106**, 4868–4884. 10.1021/cr050553y 17165678

[bb13] Wu, Y. T. & Siegel, J. S. (2006). *Chem. Rev.* **106**, 4843–4867. 10.1021/cr050554q 17165677

[bb14] Zabula, A. V., Spisak, S. N., Filatov, A. S., Rogachev, A. Y. & Petrukhina, M. A. (2011). *Angew. Chem. Int. Ed.* **50**, 2971–2974. 10.1002/anie.201007762 21404379

